# Libman-Sacks Endocarditis in a Congenital Valve Defect: A Case Report

**DOI:** 10.7759/cureus.66536

**Published:** 2024-08-09

**Authors:** Soukaina Zaher, Kawtar Nassar, Ahlam Ajerouassi, Saadia Janani

**Affiliations:** 1 Rheumatology, Ibn Rochd University Hospital Center of Casablanca, Casablanca, MAR; 2 Rheumatology, University Hospital of Ibn Rochd, Casablanca, MAR; 3 Medicine and Pharmacy, Hassan II University, Casablanca, MAR

**Keywords:** prognosis, congenital valve malformation, lupus, aseptic endocarditis, libman-sacks endocarditis

## Abstract

Libman-Sacks endocarditis (LSE) is a rare complication of systemic lupus erythematosus (SLE), characterized by noninfectious vegetation on normal heart valves. We present the case of a 20-year-old woman with SLE and a congenital valve malformation. Despite a year of effective SLE treatment, she later developed stage IV dyspnea, chest pain, and signs of right heart failure. Investigations revealed active lupus, mitral valve vegetation, agenesis of the posterior mitral leaflet, and severe mitral insufficiency. The patient was treated with corticosteroids, antibiotics, anticoagulants, and symptomatic heart failure management. Despite initial improvement, her condition deteriorated, and she did not respond to resuscitation. While LSE often responds well to treatment, severe valvulopathy, particularly with congenital valve defects, can result in fatal outcomes.

## Introduction

Libman-Sacks endocarditis (LSE), also known as nonbacterial thrombotic endocarditis, is a rare cardiac complication of systemic lupus erythematosus (SLE), characterized by noninfectious vegetation on normal heart valves, particularly the mitral valve [1]. This condition is usually asymptomatic, with a diagnosis made through echocardiography and/or histopathology. Treatment generally includes anticoagulation, corticosteroids, and sometimes surgery [2]. We present a case of LSE in a patient with lupus and a congenital heart malformation.

Note: This article was previously posted on the medRiv preprint server on April 27, 2024 [3].

## Case presentation

A 20-year-old female, deaf-mute since birth, was followed in our department for one year for SLE. She presented with joint involvement, including polyarthritis affecting large, medium, and small joints, hemolytic anemia, and immunological issues (Table 1). She was treated with corticosteroids at 20 mg per day and synthetic antimalarials, showing a good response. After one year, she presented with stage IV dyspnea and chest pain. A clinical examination revealed signs of right heart failure. Investigations showed elevated inflammatory markers and abnormal liver function tests (Table 1).

**Table 1:** Paraclinical data of the patient ANA: antinuclear antibodies; ALT: alanine aminotransferase; AST: aspartate aminotransferase; C: complement; HGB: hemoglobin; MCHC: mean corpuscular hemoglobin concentration; MCV: mean corpuscular volume; NA: not available; PCWP: pulmonary capillary wedge pressure

Parameter	Data on lupus diagnosis	Data on decompensation	Reference range
HGB (g/dl)	12.6	9.7	12.0-13.0 g/dl
MCV (fl)	87	82	80-100 fl
MCHC (g/dl)	32	32	31-36% Hb/cell
WBC (elements/mm^3^)	3,300	7,430	4,000-10,000/mm^3^
Lymphocytes (elements/mm^3^)	780	743	1,500-4,000/mm^3^
Platelets (elements/mm^3^)	319,000	442,000	150.000-400.000/mm^3^
ANA (IU/ml)	>1:160 (negative typing)	NA	<1:60 IU/ml
DNA antibodies (IU/ml)	Positive	NA	<10 IU/ml
C3 (g/l)	1.30	0.55	0.90-2.10 g/l
C4 (g/l)	0.23	0.05	0.10-0.40 g/l
C1q (mg/l)	NA	72	118-244 mg/l
CRP (mg/dl)	1.1	2.2	<0.3 mg/dl
AST (IU/ml)	23	74	6-25 IU/ml
ALT (IU/ml)	16	171	6-25 IU/ml
D-dimer (mg/l)	NA	8.05	<0.28 mg/l
PCWP (mmHg)	18	80	4-12 mmHg

**Figure 1:**
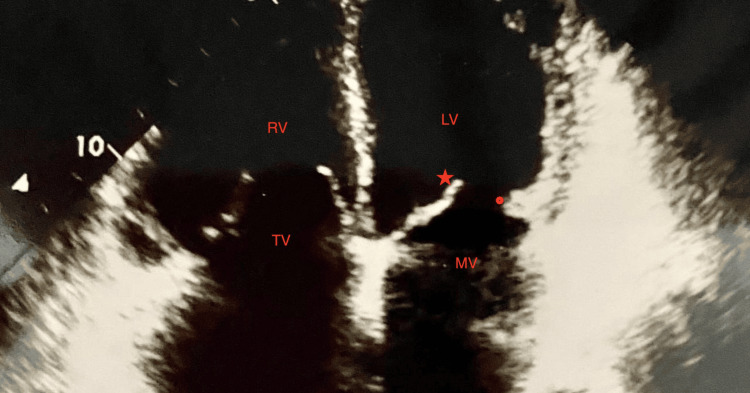
Transthoracic echocardiography showing vegetation on the mitral valve, associated with agenesis of the posterior mitral leaflet red star: vegetation; red ring: agenesis of the posterior mitral leaflet LV: left ventricle; RV: right ventricle; MV: mitral valve; TV: tricuspid valve

The lupus workup revealed consumption of complement C3, C4, and C1q. Transthoracic echocardiography showed vegetation on the mitral valve, associated with agenesis of the posterior mitral leaflet, severe mitral insufficiency, and severe pulmonary hypertension (Figure 1).

Thoraco-abdominal and cerebral CT angiography did not reveal signs of thrombosis. Screening for antiphospholipid syndrome and infectious workup were negative. The patient was started on corticosteroids at 1 mg/kg per day, antibiotics (third-generation cephalosporin combined with gentamicin), anticoagulation, and symptomatic treatment for heart failure, with an indication for valve replacement. Initially, the patient showed clinical improvement but later experienced worsening heart failure, complicated by cardiogenic shock resistant to resuscitation measures.

## Discussion

LSE is a rare but severe manifestation in patients with SLE and other autoimmune diseases. It is usually found at autopsy, with a prevalence of 0.9-1.6% [4]. LSE typically affects lupus patients aged 40-80 years, with a predilection for the mitral valve, followed by the aortic and tricuspid valves. Simultaneous involvement of two or three valves is exceptionally rare [5,6]. Patients often present with asymptomatic valvular abnormalities, making echocardiography essential for accurate diagnosis [7,8]. Among the 135 reported cases of LSE, no underlying cardiac malformation has been noted, highlighting the uniqueness of our case. The etiology of LSE involves complex immune mechanisms that lead to hypercoagulability and valve damage [1]. Diagnosis requires clinical acumen, with echocardiography being the primary evaluation method [9]. Treatment focuses on managing the underlying disease, such as SLE in this case. Corticosteroids are used to reduce inflammation but may also lead to tissue scarring and fibrosis, which can exacerbate valve damage [10,11]. Anticoagulation is considered for secondary prevention, and surgical valve replacement is recommended for severe cases. Regular follow-up is crucial to monitor disease progression [2,12].

## Conclusions

LSE typically responds favorably to treatment, except in cases of severe valvulopathy, which can be fatal. This was exemplified by our patient, who presented with decompensated heart failure due to LSE against the backdrop of a congenital heart malformation. Therefore, strict clinical and echocardiographic monitoring of lupus patients is warranted.
